# Comprehensive analysis of *Toxoplasma gondii* migration routes and tissue dissemination in the host

**DOI:** 10.1371/journal.pntd.0013369

**Published:** 2025-07-31

**Authors:** Carlos J. Ramírez-Flores, Ricardo Mondragón-Flores

**Affiliations:** 1 Department of Infectomics and Molecular Pathogenesis, Center for Research and Advanced Studies (Cinvestav). Av. IPN 2508, Col. San Pedro Zacatenco, Gustavo A. Madero, Mexico City, Mexico; 2 Department of Biochemistry, Cinvestav, Mexico; Ege Universitesi Tip Fakultesi, TÜRKIYE

## Abstract

*Toxoplasma gondii* is a highly adaptable intracellular parasite capable of infecting a wide range of warm-blooded animals, including humans. Following the ingestion of cysts and oocysts, the parasites rapidly emerge and transmigrate through the bloodstream, initiating a complex infection process. Despite reports on the parasite’s dissemination, the mechanisms behind its migration remain unclear. Recent advances using innovative 3D models and various host systems are beginning to shed light on the migratory routes and strategies employed by *T. gondii.* This review compiles current knowledge on the migration and dissemination of *T. gondii*, from its initial interactions in the gut to its invasion of immune-privileged organs. This review provides a comprehensive understanding of how *T. gondii* establishes its infection crossing the most impermeable biological barriers within the host.

## Introduction

*Toxoplasma gondii* infects nearly one-third of the global population, most often after ingestion of oocysts or tissue cysts present in water or unwashed washed produce and in undercooked meat, respectively [1]. Once acquired, *T. gondii* crosses the intestinal barrier and spreads through the bloodstream to subsequently form cysts in the brain. It can also reach the placenta and eyes, causing chronic infections, neuroinflammation, congenital toxoplasmosis and retinochoroiditis [2].

Unlike common foodborne pathogens like *Salmonella* or *Norovirus*, which primarily cause acute gastrointestinal illnesses, *T. gondii* does not remain confined to the gut: within days it actively disseminates through blood and lymph to immunologically privileged sites such as the brain, eye and placenta, where it converts to long-lived bradyzoite cysts that can persist for the host’s lifetime [3–6]. This long-term presence is facilitated by sophisticated immune evasion strategies, with *T. gondii* secreting effector proteins that modulate immune responses, allowing it to persist in immune-privileged sites like the central nervous system (CNS).

Beyond immune evasion, *T. gondii*’s presence in the CNS is associated with significant neurological consequences. The parasite alters neurotransmitter systems, such as dopamine and gamma-aminobutyric acid (GABA), a shift linked to anxiety-like behavior, depression and other psychiatric symptoms in infected hosts [7]. Infection severity depends on host genetics, parasite genotype, and the entry route. Highly virulent, non-cyst-forming type I tachyzoites are exceptionally motile and cross epithelia efficiently, whereas the cyst-forming type II and III strains migrate through epithelial barriers far less robustly [8]. These factors determine the ability of the parasite to disseminate and the severity of the disease in the host [9].

**Fig 1:**
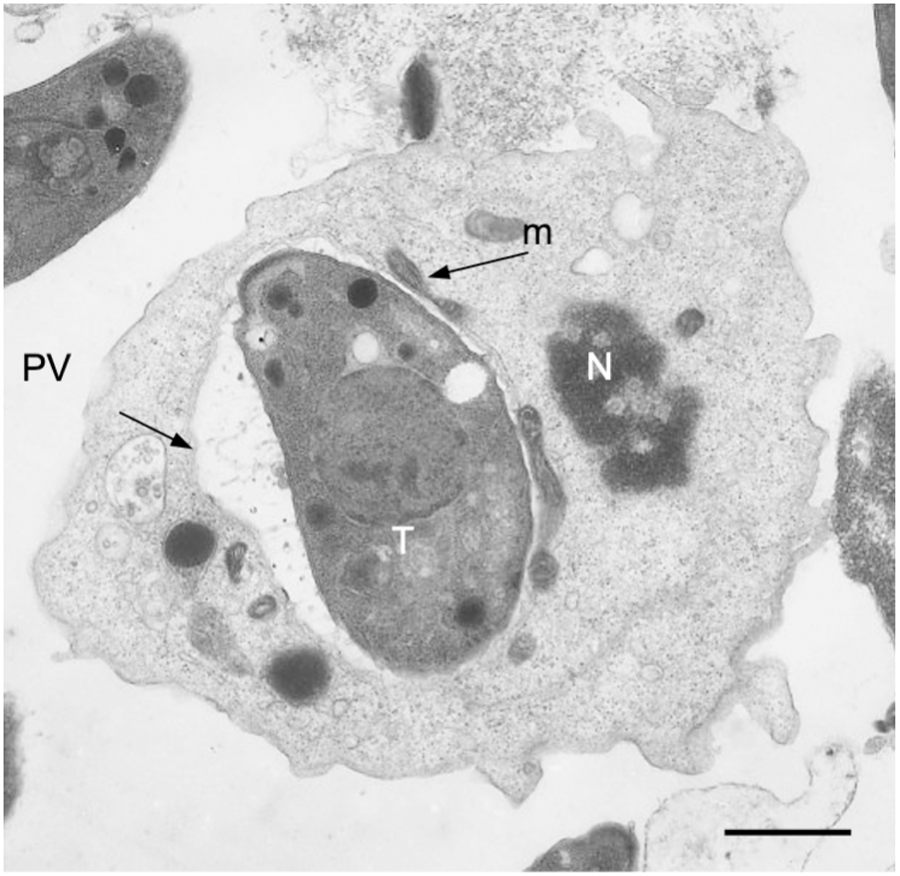
Mouse peritoneal macrophage invaded by a tachyzoite of *T. gondii.* T, tachyzoite; N, nucleus of host cell; PV, parasitophorous vacuole; m, host mitochondria. Thin section micrographed in a Transmission Electron Microscope. Scale bar = 1 μm.

**Fig 2:**
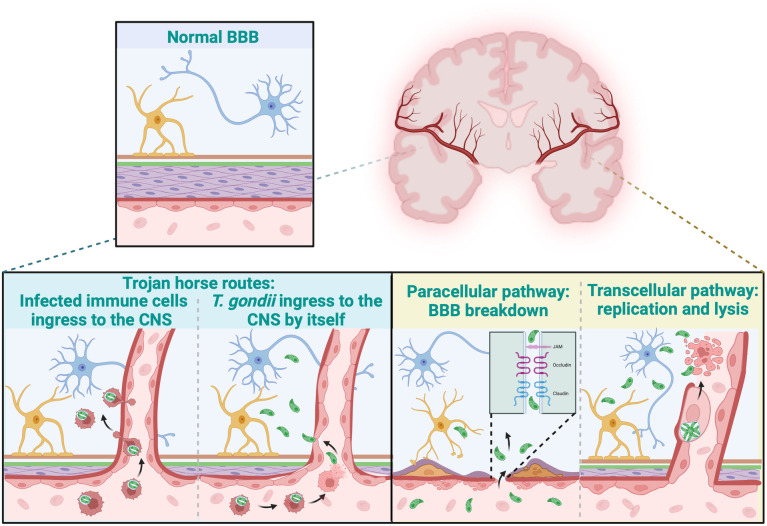
*Toxoplasma gondii* crossing the blood–brain barrier. *Toxoplasma gondii* appears to cross the blood–brain barrier (BBB) via two, probably overlapping steps. First, it hijacks phagocytic immune cells like a “Trojan-horse”, transporting the parasite to brain capillaries where it is either released nearby or transported directly through them while still hidden within migrating leukocytes. Second, once free tachyzoites contact the endothelial layer, they enter the central nervous system (CNS) by transiently loosening TJ (paracellular pathway) or by infecting and lysing endothelial cells after intracellular replication (transepithelial pathway). Figure created with BioRender.com.

Deciphering the parasite-host interactions that allow *T. gondii* to cross biological barriers—especially the blood–brain barrier (BBB)—is critical for designing both drugs and vaccines, as current evidence indicates that the parasite actively modulates host pathways to initiate acute disease and establish chronic infection [10–12]. Beyond the passive carriage implied by the “Trojan horse” hypothesis (Figs 1 and 2), in which phagocytic cells are invaded by the parasite and, due to their strong migratory capacity through chemotaxis and diapedesis, transport the parasite located within a parasitophorous vacuole, across multiple biological barriers. Instead, *T. gondii* manipulates host-cell signaling, alters tight junction (TJ) integrity, and modulates local immune responses, thereby facilitating its own translocation across physiological barriers [10]. By evading immune detection, the parasite can persist in immunologically privileged sites, like the CNS, leading to chronic infections. Understanding the cellular and molecular effectors involved in *T. gondii* migration across biological barriers and evasion of the host immune response is crucial in designing strategies to prevent the parasite from crossing the BBB, the placenta, and the retinal barrier. The following sections will explore the active mechanisms underlying these processes.

### Uncovering the unique features of *T. gondii*

Like *T. gondii*, numerous pathogens not only affect the site of infection but also migrate to other organs, causing systemic disease. These pathogens share the ability to manipulate host defenses to facilitate their spread. Well known examples are *Salmonella enterica* [13,14], *Listeria monocytogenes* [15], *Entamoeba histolytica* [16–19] and the Apicomplexans *Cryptosporidium* spp [20] and *Plasmodium* spp. [21]. They modify the integrity of epithelial barriers, disrupt TJ, alter mucus production, and interact with connective tissue, reaching vascular tissue enabling them to cross biological barriers. This capacity to disseminate beyond the initial site of entry highlights the complex mechanisms pathogens use to evade immune responses and establish chronic infections, leading to complications in various organs.

*Toxoplasma* stands out among these pathogens due to its ability to establish long-term latent infections and interact uniquely with the host’s immune system. *T. gondii* forms tissue cysts in immune-privileged sites like the brain, eyes, and placenta, allowing it to persist for the host’s lifetime [4–6]. *T. gondii* is highly neurotropic, often infecting the CNS, where it can disrupt neuronal function and contribute to behavioral changes, such as increased anxiety and depression, which is not commonly seen in other ingested pathogens. *T. gondii* can manipulate the behavior of murine hosts by affecting basic instincts such as the loss of fear of feline predators, thus facilitating transmission to its definitive feline host [22]. It also influences the behavior of humans, although further research is needed on this subject (topics explained below). Its unique life cycle, involving both definitive (feline) and intermediate (non-feline) hosts, and its ability to invade immune-privileged sites, where it remains undetected, distinguish it from other pathogens.

*T. gondii* exploits three, partly overlapping transport routes whose relative importance changes depending on the parasite stage and host context. Paracellular gliding is most prominent during the first 24–48 h of acute infection: highly motile tachyzoites of type I strains traverse intestinal and endothelial TJ in a motor-driven, MIC2/ICAM-1–dependent manner [8]. Concurrently, the parasite deploys a “Trojan-horse” strategy, converting infected dendritic cells (DC) and monocytes into hypermigratory carriers that transport intracellular tachyzoites via lymph and blood; this route becomes especially critical for systemic spread in less motile type II/III strains and during reactivation in immunocompromised hosts [23,24]. A third, less common transcellular pathway—direct penetration and replication in endothelial cells—has been visualized in cortical capillaries during early brain invasion and may become relevant when inflammatory signals disrupt barrier integrity [25]. Once chronic cysts form, stage conversion events are largely confined to immune-privileged tissues (brain, retina, placenta), and renewed dissemination depends on local cyst rupture rather than long-distance migration.

**Fig 3:**
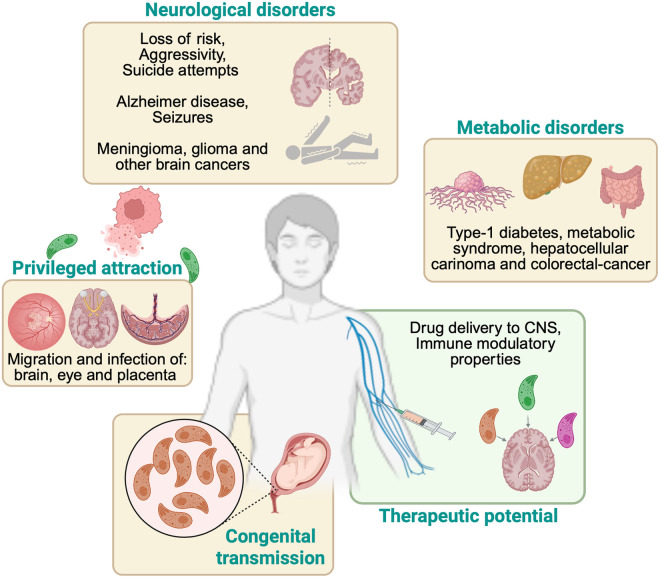
*Toxoplasma gondii* and its reported human sequelae or its potential associated manifestations. The silhouette represents a gender-neutral individual to emphasize its relevance at the population level. This ubiquitous intracellular parasite disseminates beyond the intestine and establishes chronic cysts in multiple organs. Epidemiological studies link latent infection to metabolic disorders (higher seropositivity in type 1 diabetes) [87,88] and a 1.7-fold increased risk in metabolic-syndrome [89] and to several solid tumors, such as glioma [90], colorectal cancer [93] and hepatocellular carcinoma [94]. *T. gondii* remains a major cause of congenital neuroocular disease [100,111] and, in immunocompromised hosts, of encephalitis [64]. Its remarkable tissue-invasive characteristics are also being repurposed in genetically modified “live-vector” strains for drug delivery and tumor immunotherapy [26]. Figure created with BioRender.com.

These features make *T. gondii* a complex pathogen, highlighting the importance of understanding its ability to evade immune cells and cross biological barriers for developing effective therapies. Recent findings have explored how *T. gondii* can be engineered to deliver large therapeutic proteins directly into neurons, utilizing its ability to cross the BBB, potentially revolutionizing treatments for neurological disorders (Fig 3) [26]. This technique has shown promising results for treating neurological diseases such as Alzheimer’s and Parkinson’s by delivering proteins directly to brain cells [26–28] (addressed in a section below).

**Toxoplasma*’*s neurotropism allows it to invade the CNS by disrupting the BBB, a critical step in the pathogenesis of chronic infection [29]. This disruption enables the parasite to enter the CNS, where it forms cysts that remain in an immunologically silent state, primarily within neurons [30]. These cysts contribute to sustained neuroinflammation and microvascular dysfunction [31]. The inflammatory response is marked by the activation of glial cells, including astrocytes and microglia, and the upregulation of cytokines such as tumor necrosis factor-α (TNF-α) and various chemokines, which further disrupt the BBB and facilitate immune cell infiltration into the brain [32]. Chronic neuroinflammation induced by *T. gondii* infection has been linked to behavioral changes and an increased risk of neuropsychiatric disorders, including anxiety, depression, and schizophrenia [32,33]. These alterations are thought to result from the parasite’s impact on neurotransmitter systems and immune-mediated neuronal damage.

### Uncovering the migratory and infection profiles of the three canonical *Toxoplasma gondii* lineages

Population‐genetic analyses show that majority of *Toxoplasma gondii* isolates circulating in North America and Europe belong to three dominant clonal lineages—type I, type II, and type III—originally defined by multilocus restriction fragment length polymorphism (RFLP) typing of surface antigens and metabolic genes, and subsequently formalized in a high-resolution PCR-RFLP panel; each lineage possesses a characteristic composite haplotype, stable across all loci, that correlates with distinct virulence and epidemiological profiles [34–36].

Early comparative work established that the highly virulent type I lineage (e.g., RH, GT1) is a “hypermigrator.” *In vitro* assays on polarized epithelial monolayers and Matrigel, as well as in *ex-vivo* mouse-ileum chambers, showed that type I tachyzoites cross barriers three logs more efficiently than type II (ME49/Pru) or type III (CTG/VEG) parasites; a long-distance migration phenotype (>110 µm in agarose) is expressed by virtually all type I isolates but is absent in types II and III [8]. These intrinsic differences are mirrored in the “Trojan-horse” pathway. When DCs are infected *in vitro*, tachyzoites of all three lineages trigger a hypermigratory shift, but type I-infected DCs move faster and carry a higher parasite load; however, both type II and III strains can exploit this transport mechanism, highlighting a quantitatively variable but lineage-independent utilization of host leukocytes [37].

Mechanistically, strain-specific alleles of secreted rhoptry and dense-granule kinases regulate motility and survival. Type I parasites possess active ROP18/ROP5 complexes that neutralize murine IRGs, as well as efficient processing of the MIC2 adhesin, which enhances gliding motility and barrier crossing, whereas type II and III alleles confer slower movement but improved cystogenesis and long-term persistence *in vivo* [38].

Collectively, the published data present a gradient: type I strains combine hypermotility with acute virulence and rapid systemic spread; type II strains display intermediate motility and cause chronic, cyst-rich infections; type III strains are the least motile and virulent, relying more on leukocyte-mediated transport than direct paracellular migration. Understanding how these lineage-specific attributes intersect with host immunity clarifies why disease severity and dissemination routes vary so greatly among *T. gondii* infections.

### Uncovering the sites of infection in the intestine: Everything starts in the gut

Following ingestion, *T. gondii* cysts and oocysts encounters the acidic environment of the stomach, where pepsin together with the bile salts play a crucial role in breaking down the protective walls of cysts and oocysts [39]. The release of bradyzoites from tissue cysts or from sporozoites originating from oocysts allows interaction with the small intestine and invasion of host cells. In addition to its function during excystation, pepsin also acts as an activator of bradyzoites after their exteriorization, which increases the capacity of bradyzoites to infect human cells and human-like intestines *in vitro* [40]. In addition, peptidases like trypsin also has an effect activating the motility of tachyzoites [41].

Once the cyst wall is digested in the intestinal lumen, many researchers hypothesize that the released bradyzoites invade enterocytes, rapidly differentiate into tachyzoites, and then initiate systemic dissemination [42,43]. However, definitive evidence remains scarce because it is technically difficult to capture replicating parasites in the intestine shortly after ingestion. In Dubey’s pioneering studies in the 90s, parasites were detected in the lamina propria of mice within hours of oral exposure to oocysts or tissue cysts, with only brief contact in enterocytes [44,45]. Those experiments relied solely on electron microscopy and lacked stage-specific markers, leaving the developmental state of the parasites uncertain. Recent work has addressed this gap by using fluorescently labelled parasites in orally infected mice and tracking them using flow cytometry and intravital multiphoton microscopy, allowing real-time visualization of migration from the intestinal mucosa [46]. In addition to the mouse model, new technologies such as gut-on-a-chip have helped to elucidate this process (discussed in a later section) [40]. As soon as three days post-infection, parasites localized in small foci within the lamina propria, with macrophages, neutrophils, and monocytes being the primary infected immune cells, suggesting an eventual parasite migration in the “Trojan horse” (Figs 1 and 2) [46]. By six days post-infection, larger foci of replicating parasites appeared, and viable tachyzoites were found in the intestinal lumen, potentially serving as a reservoir promoting continued intestinal dissemination and suggesting a mechanism for luminal spread.

The use of animal models in *T. gondii* infection research, particularly in studying gut infections, has faced several challenges, such as: disruption of the tissue by the mechanical force required to isolate the intestine, limited sample amounts, irregular infection sites, and low parasite burden, all of which can complicate data interpretation [40]. However, recent *in vitro*, *in vivo*, and *ex vivo* experiments have provided valuable insights into the early interactions of *T. gondii* with the host [40]. These studies reveal that the jejunum is the preferred site of infection in orally infected mice [40,46]. By five days post-infection, foci of infection are evenly distributed across small areas in the small intestine [40]. Additionally, extracellular parasites demonstrate the ability to transmigrate actively, likely through a paracellular pathway, to locate within the intestinal stroma, in *in vivo* and *ex vivo* jejunal tissue [40]. This suggests a more complex and dynamic process of infection than previously understood. Contrary to what was previously accepted, new analyses have further demonstrated that *T. gondii* preferentially transmigrates through the enterocytes, with limited infection and replication inside the enterocytes themselves followed by 3 and 5 days post oral infection in mice. By three days post-infection, most parasites were found in the stroma, actively replicating [40]. Supporting the idea that the stroma is the critical site for parasite replication, progression and interaction with the host immune cells.

### Uncovering the intestinal bradyzoite-to-tachyzoite transition process

*Toxoplasma gondii* orchestrates its rapid growth and ability to switch between acute (tachyzoite) and latent (bradyzoite) forms through extensive epigenetic regulation, rather than fixed genetic changes. By flexibly adding or removing chemical marks on histones and deploying stage-specific transcription factors, the parasite can rapidly reprogram large sets of genes in response to environmental cues, synchronizing cell cycle timing with developmental decisions. This reversible “histone code” along with epigenetic mechanisms, signaling pathways, and cell cycle control, offers a master control that underlies *T. gondii*’s adaptability within the host, highlighting chromatin-based processes as attractive targets for novel therapeutic strategies [47,48]. (I) Epigenetic control: chromatin states influence gene expression, with transcription factors like TgAP2XI-4 playing a key role in the switch. Knockout of TgAP2XI-4 alters gene expression and reduces cyst formation [49]. Furthermore, Waldman and colleagues identify Bradyzoite-Formation Deficient 1 (BFD1) as the transcription factor at the top of the bradyzoite differentiation hierarchy [50]. Loss of BFD1 abolishes cyst formation, whereas its conditional expression is sufficient to drive tachyzoites into the bradyzoite program even in the absence of stress, establishing BFD1 as a genuine master regulator of stage conversion [50]. (II) Stress responsive signaling: alkaline pH, nutritional stress or IFN-γ raise cAMP/Ca^2+^ and activate TgPKAc3; deletion of this kinase accelerates bradyzoite differentiation [51]. (III) Cell cycle checkpoint: regulators such as TgCrk2 determine whether the parasite continues replication or differentiates into bradyzoites. TgCrk2 imposes a late “restriction” point in G1: deletion of TgCrk2 traps parasites in G1 and favors conversion [52].

While the molecular mechanisms are understood, the precise timing of the process is still unclear. Most studies on *T. gondii* infection have been conducted in animal models, but the study of human infection remains severely limited. These animal models have provided important insights, often based on morphological changes and parasite differentiation from the bradyzoite form to the tachyzoite form mainly at the lamina propria rather than inside enterocytes. In a recent research report, tachyzoite-specific markers such as SAG1 were used by collecting parasites from the jejunum of infected mice. Three days after infection, only SAG1-positive parasites, i.e., tachyzoites, were found, suggesting a rapid switch between the two forms of *T. gondii* within the first three days [40]. Additionally, the analysis of infected intestines indicated that the switch likely occurs directly in the stroma of the intestine, as the frequency of enterocyte infection remained low [40]. These findings provide valuable insights into the timing and dynamics of *T. gondii*’s developmental transition, highlighting the importance of the stroma in parasite stage conversion.

### Uncovering the paracellular migration of *T. gondii* through epithelial and endothelial barriers

*T. gondii* is capable of breaching biological barriers and disseminating throughout the host. While the “Trojan horse” mechanism—where the parasite hijacks immune cells for transport—is well-established (Figs 1 and 2), emerging evidence suggests that *T. gondii* can also migrate extracellularly through epithelial layers and connective tissues [39,40,46]. This alternative dissemination strategy involves the degradation of intercellular junctions and alterations of the extracellular matrix (ECM), leading to increased tissue permeability and immune system modulation.

The interaction of *T. gondii* with host tissues involves significant morphological changes and modifications to TJ and adherens junction (AJ) proteins, which facilitate its dissemination through epithelial and connective tissues. The parasite secretes proteases that degrade key intercellular junction proteins in a epithelial cell model, including ZO-1, occludin, claudin-1, and E-cadherin, increasing tissue permeability and promoting paracellular migration [12]. This mechanism could be critical for the traversal of biological barriers, including the intestinal epithelium, retinal pigment epithelium, and BBB (Fig 2).

In the intestinal epithelium, *T. gondii* infection leads to a loss of barrier function, as evidenced by decreased transepithelial electrical resistance (TEER) and altered expression of TJ proteins such as occludin and ZO-1, ultimately increasing mucosal permeability and facilitating parasite dissemination [53]. Similarly, in the retinal pigment epithelium (RPE cells), *T. gondii* disrupts TJ integrity, increasing paracellular permeability and contributing to retinal injury [54]. As observed in other epithelial cells models, *T. gondii* infection alters the distribution of TJ proteins, resulting in decreased TEER and compromised barrier function [12]. Moreover, the parasite’s ability to disrupt TJ pathways at the BBB compromises barrier integrity, allowing neuroinvasion and persistence within the CNS [10]. These processes collectively enable *T. gondii* to spread efficiently throughout the host, establishing infection in immunoprivileged organs (Figs 2 and 3).

Beyond epithelial tissues, *T. gondii* modulates endothelial function, particularly at the BBB, to invade the CNS. Infection triggers upregulation of adhesion molecules such as ICAM-1 and VCAM-1 on brain endothelial cells, promoting vascular permeability and inflammation [55]. Additionally, platelet-fibrin clot formation within cerebral vessels reduces blood flow and contributes to BBB disruption. Microvascular dysfunction further exacerbates permeability changes, with capillary rarefaction, altered vasodilation responses, and decreased angiogenesis collectively weakening the BBB [31].

Endothelial cells in mice choroid plexus are invaded by *T. gondii* before the infection of the BBB, leading to an upregulation of IFN-γ, TNF-α, IL-6 [56]. Additionally, *T. gondii* infection of the choroid plexus is associated with the upregulation of metalloproteases MMP-8 and MMP-13 [56], suggesting that the parasite infection influences the ECM by altering collagen distribution and disrupting barrier integrity. These processes, combined with endothelial activation and microvascular dysfunction, facilitate *T. gondii* translocation across the BBB, allowing it to establish infection within the CNS [10,56]. Overall, the interplay between *T. gondii*–induced epithelial and endothelial barrier disruption, microvascular alterations, and ECM remodeling underscores the parasite’s sophisticated mechanisms for systemic dissemination and neuroinvasion. The potential involvement of *T. gondii* secreted proteases and molecules in the direct degradation of ECM components is an emerging area of investigation, warranting further exploration to elucidate their role in barrier disruption.

### Uncovering an active migration through the connective tissue

It is accepted that *T. gondii* disseminates through the “Trojan horse” mechanism, where it infects host immune cells such as monocytes and DC to facilitate its spread throughout the body and reach immunoprivileged organs (Fig 1) [57,58]. This strategy enables the parasite to travel not only through the bloodstream but also to move within and across various biological barriers, including epithelial, endothelial, and connective tissue. *In vivo* and *ex vivo* studies demonstrate that the “Trojan horse” pathway is considerably more efficient for long-distance spread than free tachyzoites release and simultaneously protects the parasite from early immune attack, advantages that favor establishment of chronic infection. Mice injected with DCs harboring intracellular tachyzoites accumulated 2- to 25-fold higher parasite burden in the spleen and mesenteric lymph nodes within 16 h than their littermates receiving the same number of free parasites, an effect more pronounced in the less motile type II and III strains [23].

*T. gondii* induces a hypermigratory phenotype in infected DC, enhancing motility and altering signaling pathways that support tissue migration [59]. *T. gondii* infection triggers a mesenchymal-to-amoeboid transition in DC, promoting rapid movement, which is mediated by GABAergic signaling, calcium fluxes, and the activation of the MAPK Erk signaling cascade [60]. Moreover, the parasite secretes effector proteins like GRA15 and GRA24, which modulate host cell signaling pathways, such as NF-κB and p38 MAPK, to further enhance the migratory capacity of infected macrophages [61]. This ability to manipulate immune cells and cross biological barriers is essential for *T. gondii* to invade immunoprivileged organs, including the brain, where it can establish chronic infection and evade immune clearance.

Infected DC undergo a mesenchymal-to-amoeboid transition, enhancing their motility through the ECM. This transition is characterized by integrin-independent migration, enabling DCs to traverse a three-dimensional collagen matrix without substantial degradation [62]. Amoeboid migration is facilitated by the dissolution of podosome structures and the secretion of tissue inhibitor of metalloproteinases-1 (TIMP-1), which reduces pericellular proteolysis and limits ECM remodeling [59]. Parasite migration that proceeds without proteolytic degradation is crucial for efficient dissemination, allowing infected cells to navigate connective tissues without extensive matrix degradation.

Recent studies have demonstrated that *T. gondii* can survive extracellularly in the connective tissue and actively migrate through epithelial barriers via paracellular migration, independent of immune cells [40]. Since the first micrographs generated by J.P. Dubey in the 1990s, evidence has suggested the presence of extracellular parasites in the connective tissue of orally infected mice [44]. A decade ago, Gregg and colleagues discussed the existence of viable extracellular parasites in the intestinal lumen, potentially contributing to the dissemination of the parasite within the gut [46]. The modification of the host cell by *T. gondii* infection leads to the upregulation of MMPs, as previously discussed, consequently facilitating its spread [56]. Recent studies have provided direct evidence of extracellular *T. gondii* migrating and moving through collagen- and fibronectin-rich 3D platforms, further supporting the concept of active, immune-cell-independent dissemination [40].

*T. gondii* infection affects the ECM by altering collagen dynamics. *T. gondii* infection of the choroid plexus is associated with the upregulation of metalloproteases MMP-8 and MMP-13 in mouse [56], both collagenases which activity may influence ECM remodeling. In the jejunum, infection leads to changes in collagen deposition, with a reduction in type I collagen fibers and submucosa thickness, indicating ECM disorganization. This structural damage is associated with increased expression of ICAM-1 and serotonin, which may play roles in the immune response and tissue remodeling during infection [63].

### Uncovering the translocation of *T. gondii* to the brain

According to the Centers for Disease Control and Prevention, toxoplasmic encephalitis remains the most common intracerebral mass lesion in people with AIDS and other severe immunosuppressive states [64]. A 2017 *Lancet HIV* meta-analysis of 74 studies estimated that 35.8% of people with HIV are coinfected with *T. gondii*, equivalent to 13 million people worldwide, 87% of whom reside in sub-Saharan Africa [65]. The initial translocation of *T. gondii* to the brain parenchyma primarily occurs through cortical capillaries [29]. This process is initially constrained by the integrity of the BBB, which restricts parasite transit. However, localized increases in BBB permeability can occur adjacent to replicative parasite foci, facilitating CNS invasion. *T. gondii* employs poorly understood mechanisms to cross the BBB, significantly affecting endothelial cell function, adhesion molecule expression, and neuroinflammation [66]. The parasite can modulate the expression of adhesion molecules such as ICAM-1 on brain endothelial cells, a process further enhanced by IFN-γ, thereby promoting its passage through the BBB [67]. This modulation is part of a ‘Trojan horse’ strategy, wherein *T. gondii* infects antigen-presenting cells, particularly CD11b(+)/CD11c(−) cells, which then migrate across the BBB [67]. Additionally, *T. gondii*-infected DC undergo a migratory switch from integrin-independent to integrin-dependent motility upon contact with brain endothelial cells. This switch is crucial for the transendothelial migration of these cells and is mediated by β1 and β2 integrins and ICAM-1 [67]. The dysregulation of focal adhesion kinase (FAK) in endothelial cells upon *T. gondii* infection further facilitates parasite translocation across polarized brain endothelial monolayers by altering TJ stability [68]. Moreover, *T. gondii* can induce inflammatory responses in cortical micro blood vessels, exacerbating BBB permeability and promoting parasite translocation, with Rab13 upregulation being identified as a potential mechanism that regulates BBB permeability and enhances CNS invasion [10].

Recent *in vivo* imaging showed that *T. gondii* deploys a two-step strategy to seed the brain. First, the parasite enters DCs, the Trojan horse—to reach the brain microvasculature: intracarotid injection of *T. gondii*-infected DCs into mice revealed that ICAM-1/CD18 adhesion arrests these parasitized leukocytes within cortical capillaries. Once DCs extravasate, extracellular tachyzoites are released that immediately invade nearby neurons [69]. Second, crossing of the BBB is accomplished by the free-tachyzoites themselves, not by their cellular carriers. How they traverse intact endothelium is still unresolved, but complementary work shows that tachyzoites can induce host TIMP-1 via a GRA24-p38-MAPK axis to glide non-disruptively across polarized monolayers of brain endothelial [70].

Once inside the CNS, *T. gondii* establishes persistent tissue cysts predominantly within neurons [3]. These cysts are immunologically silent and can persist throughout the host’s lifetime. However, their presence is associated with sustained neuroinflammation, characterized by microglial and astrocytic activation and the production of pro-inflammatory cytokines such as TNF-α and IFN-γ [31,32]. Long-term survival is enhanced by secreted effectors that suppress intrinsic cellular immunity: the ROP5/ROP18 kinase pair phosphorylates and inactivates IFN-γ–induced IRG GTPases [71], while the dense-granule protein TgIST recruits host chromatin repressors to inhibit STAT1-dependent gene expression [72]. Junctional disruption for entry, immunevasive effectors, and cyst formation within immune-privileged neuronal cells underpin parasite persistence in the CNS.

In the context of long-term infection, several studies have reported significant behavioral alterations linked to neuroinflammation and BBB disruption (covered in the next section). The study by Castaño Barrios and colleagues [32] demonstrated a neuroinflammatory response characterized by the upregulation of pro-inflammatory cytokines such as TNF-α and CC-chemokines such as CCL2/MCP-1 in brain tissue, as well as increased levels of IFN-γ, TNF-α, and CCL2/MCP-1 in peripheral blood, suggesting a systemic inflammatory response that may further contribute to BBB disruption [32].

The inflammatory response to *T. gondii* infection can further compromise BBB integrity. Treatments that modulate inflammation, such as hydrocortisone, have been shown to affect BBB permeability and alter parasite loads in the brain parenchyma, underscoring the role of inflammation in facilitating parasite translocation [29]. Additionally, *T. gondii*’s interaction with endothelial cells can lead to microvascular dysfunction, including decreased cerebral blood flow, capillary rarefaction, and reduced vasodilation, all of which further weaken the BBB and contribute to neurodegenerative processes [31]. The chronic inflammatory state induced by *T. gondii* infection is thought to drive these behavioral changes, potentially through mechanisms involving neuronal damage and neurotransmitter dysregulation. The dysregulation of focal adhesion kinase (FAK) further destabilizes TJ in brain endothelial cells, enhancing BBB permeability and promoting parasite translocation [68].

Overall, *T. gondii* ability to cross the BBB, establish latent brain cysts, and trigger neuroinflammation plays a critical role in its pathogenesis within the CNS. The persistence of parasite cysts induces a chronic inflammatory environment that disrupts BBB integrity and contributes to significant behavioral alterations in infected hosts (covered in the next section), highlighting the complex interplay between parasitic infection, immune response, and neurological function.

### Uncovering the neuro-alterations caused by *T. gondii* infection

*T. gondii* infection induces significant changes in the behavior of hosts, for example in infected mice, the survival instinct against predators such as felines is inhibited with the loss of fear [73]. This phenomenon, often referred to as “fatal attraction,” involves a reduction in the innate fear of cat odors, increasing the likelihood of predation by felids, the parasite’s definitive hosts [73,74]. This behavioral manipulation is considered an adaptive strategy to favor the transmission of *T. gondii* to the definitive host involved in the sexual reproduction of the parasite. Infected mice display a consistent behavioral syndrome characterized by reduced anxiety, heightened exploratory drive, and a broad loss of predator aversion that extends beyond felid odors to include cues from foxes and rats, increasing their overall predation risk; notably, these changes occur without measurable deficits in sociability or short-term memory [22,75]. The severity of these behavioral changes scales with brain cyst burden: parasites of attenuated virulence (ASP5-KO, MYR1-KO, MyoJ-KO) or the related apicomplexan *Neospora caninum*—all of which establish far fewer cysts—produce correspondingly mild phenotypes. RNA-seq of whole brains links high cyst burdens to sustained upregulation of immune and cytokine signaling pathways, down-modulation of neuronal-synaptic genes, and elevated plasma IFN-γ/IL-12p40 levels, indicating that chronic neuroinflammation drives the behavioral syndrome [22].

The mechanisms driving these behavioral changes include disruption of the BBB, neuro-inflammation, and neurotransmitter imbalance. Infection upregulates proinflammatory cytokines such as TNF-α and IFN-γ and chemokines such as CCL2/MCP-1, CCL3/MIP-1α, and CCL5/RANTES, while downregulating the astrocytic glutamate transporter, GLT-1. The resulting increase in extracellular glutamate causes loss of dendritic-spine and synaptic dysfunction that likely rewires circuits governing fear and risk assessment [76,77].

In humans, latent toxoplasmosis has been linked to neuropsychiatric disorders such as schizophrenia, bipolar disorder, major depression, and an increased prevalence of suicide attempts [78]. The mechanistic basis of these links remains speculative; contributing factors such as altered dopamine and glutamate signaling, mild neuroinflammation, and oxidative stress have been proposed, but clear causality has not been demonstrated. Epidemiological meta-analyses further suggest a possible connection with Alzheimer’s disease, though evidence remains inconclusive and requires further investigation [79]. More established, however, is the link between *T. gondii* seropositivity and increased suicide risk, with findings indicating higher prevalence among individuals with a history of suicide attempts [80,81]. This association may stem from the parasite’s influence on decision-making and impulsivity, although the precise biological pathways remain to be elucidated.

Latent *T. gondii* infection in humans has been linked to subtle psychomotor slowing— initially documented as significantly longer reaction times in seropositive volunteers [82]—and consequently to an increased risk of real-life accidents. A large Czech case–control study found a 2.65-fold increased risk of traffic accidents among seropositive drivers [83]. A prospective cohort of 3,890 military recruits replicated this association and found RhD-negative infected individuals to be at highest risk [84]. A 2018 meta-analysis of ten independent datasets estimated a pooled odds ratio of approximately 2.0 for traffic accidents in infected subjects [85]. These findings suggest that subtle psychomotor impairments (slower reaction times, increased impulsivity, and greater risk-taking) translate the parasite’s neurobiological effects into real-world safety risks.

In summary, rodent studies show that *T. gondii* can induce pronounced, albeit partly transient, behavioral changes dependent on neuroinflammatory tone and BBB integrity, whereas human data are still limited to associative studies. Recent research in rats even reported that neuroinflammation caused by infection remits within three months despite persistent cysts [86], underscoring the need for long-term, human-focused research before firm conclusions about psychiatric risk can be drawn. Taken together, these observations illustrate a complex parasite–host neurobiology that may influence public-health outcomes, but they also highlight important knowledge gaps regarding chronic and clinically relevant effects in people.

### Uncovering systemic dissemination to metabolic tissues and its potential associated manifestations for human health

Following its excysting in the gut, *T. gondii* disseminates systemically by hijacking DC and monocytes in a “Trojan-horse” fashion [23], gliding paracellularly along the vascular endothelium [24], and occasionally replicating within endothelial cells and lysing them to cross transcellularly [25]. These complementary pathways spread to metabolically active peripheral organs such as the liver, skeletal muscle, adipose tissue and pancreatic islets, where bradyzoite cysts establish chronic low-grade inflammation. Although direct evidence of *T. gondii* localization in human metabolic tissues is still lacking, several epidemiological studies have reported statistical relationships between toxoplasmosis seropositivity and the severity of various metabolic disorders.

Two meta-analyses encompassing over 4,000 participants report a significantly higher *T. gondii* seroprevalence in individuals with type 1 diabetes [87,88]. In a cross-sectional cohort of 83 Egyptian adolescents with obesity, 57% of seropositive subjects met the International Diabetes Federation definition of metabolic syndrome compared with 33% of seronegative patients [89]. These findings suggest, but do not prove, that chronic *Toxoplasma* infection could be an independent risk modifier.

Latent *T. gondii* infection has also been epidemiologically linked to solid tumors. Prediagnostic seropositivity doubled the risk of glioma in two large U.S./Nordic cohorts [90] and was also increased in Korean patients with brain tumors [91]. A 2022 meta-analysis confirmed a significantly increased risk of brain tumors associated with *T. gondii* infection or exposure, including gliomas and meningiomas [92]. Outside the CNS, seropositivity was significantly higher in patients with colorectal-cancer in China [93] and was higher in Egyptian hepatocellular carcinoma cases than cirrhotic controls [94]. Th1 inflammation and parasite-driven STAT3/NF-κB signaling are proposed pro-tumor mechanisms, underscoring the need for prospective, strain-resolution studies [95,96]. Additional research directly examining the presence of *T. gondii* in human tumor samples is needed, even though we recognize the practical and ethical constraints that limit access to such samples.

Although mechanistic insight still largely derives from animal work—β-cell invasion, for example, impairs insulin secretion in mice [97]— consistent epidemiological associations in humans justify prospective studies to establish causality. Taken together, these lines of evidence point to a biologically plausible link that merits rigorous investigation.

### Uncovering *Toxoplasma gondii*’s therapeutic potential

#### Engineered protein delivery across the blood–brain barrier.

The obligate intracellular lifestyle and pronounced neurontropism of *T. gondii* have recently been engineered to create a “living-vector” platform that overcomes the BBB and the size limitations of viral carriers. By fusing therapeutic macromolecules (>100 kDa) with two native secretory effectors—the rhoptry transporter Toxofilin and the dense granule protein GRA16—Bracha *and colleagues* created a dual-secreting strain that delivers fully functional cargo such as Cre recombinase and full-length MeCP2 into host-cell nuclei across cultured neurons, human brain organoids, and, following a single intraperitoneal injection, into the entire murine brain. The parasites disseminated at millimeter scales within the parenchyma but did not cause any overt pathology, demonstrating efficient systemic delivery of large proteins and highlighting the translational promise of *T. gondii* as a size-unrestricted vector for the treatment of CNS disorders refractory to conventional biologics [26].

#### Live-attenuated strains as *in situ* cancer vaccines.

Independently, an attenuated, non-replicating uracil-auxotrophic *T. gondii* (NRTUA) strain has emerged as a potent immunoadjuvant against pancreatic ductal adenocarcinoma (PDAC). In a mouse model with subcutaneous Pan02, NRTUA monotherapy reduced tumor growth by invading intratumoral DC, activating MyD88/NF-κB signaling, and generating high levels of IL-12 that chemoattracted and primed *de novo* infiltrates of cytotoxic CD8⁺ T cells. When combined with anti-PD-1 blockade, NRTUA transformed the immunologically “cold” PDAC microenvironment into a “hot,” state of T-cell inflammation: the combination synergistically suppressed tumor volume and weight, boosted tumor-specific IFN-γ production, and simultaneously depleted polymorphonuclear and monocytic myeloid-derived suppressor cells. Functional ablation of CD8⁺ T cells or IL-12 abrogated these benefits, underscoring the DC-IL-12–CD8⁺ T-cell circuitry dependency. Collectively, the work positions attenuated *T. gondii* as a tractable biologic platform for reprogram the PDAC tumor microenvironment and overcome intrinsic resistance to PD-1 checkpoint inhibition [98].

#### Self-amplifying vectors for heterologous vaccination.

A third line of investigation takes advantage of *T. gondii*’s broad host spectrum, and strong Th1-skewing immunogenicity to deliver non-parasite antigens. In early proofs of concept, RH strain expressing yellow-fluorescent protein was engineered; single subcutaneous doses generated robust IFN-γ–driven cellular immunity and partial cross-protection against *Eimeria* infection in both resistant and susceptible hosts. More recently, cyst-deficient or gene-deleted strains such as PruΔgra72 have induced sterilizing protection against acute and chronic toxoplasmosis while remaining avirulent, illustrating a blueprint for “plug-and-play” vaccine chassis that present exogenous antigens via both MHC-I and -II pathways for hard-to-vaccinate pathogens or tumors [99].

Together, these studies reposition *T. gondii* from a model pathogen to a versatile biotransport vehicle, capable of overcoming biological barriers, reversing tumor immune evasion, and serving as a live vector for complex antigens. This emerging toolkit could complement, rather than replace, conventional nanocarriers and viral platforms in next-generation therapies. However, its propensity to form permanent cysts in immunologically privileged tissues and to reactivate or cause congenital and ocular diseases cautions that any therapeutic application will require rigorous attenuation, fail-safe genetic switches, and robust preclinical safety testing to balance the possibilities against potential risk.

### Uncovering the migration of *T. gondii* to the retina

*Toxoplasma gondii* infection in the eye, known as ocular toxoplasmosis (OT), is a significant cause of posterior uveitis in 20–40% of all cases and can lead to vision impairment due to retinitis, often accompanied by vitritis and choroiditis [100]. The prevalence of OT is higher (up to 6%) in Latin-America and other middle-income regions [101]. While previous studies in human retinal cell culture have suggested the modification of the TJ during an active process of tachyzoite transmigration, as previously discussed [54]. In most immunocompetent adults OT represents reactivation of congenital or acquired tissue cysts lodged in the retina. The conversion from bradyzoite to tachyzoite triggers a focal necrotizing retinochoroiditis with overlying vitritis, driven by local Th1 cytokines (IFN-γ, IL-12) and mononuclear infiltrates that attempt to block parasite replication [102].

The animal model for studying the ocular toxoplasmosis via infection of tachyzoites in mice was recently established and has proven to be challenging [103,104]. Research into how *Toxoplasma gondii* accesses the retina has identified several potential pathways, though definitive evidence remains limited. It is suggested that the parasite may cross the blood–retinal barrier (BRB) through transport mediated by infected leukocytes including DC and monocytes, which migrate across the retinal endothelium, to deliver the parasite to retinal tissue [105]. Additionally, *T. gondii* tachyzoites might traverse the spaces between retinal endothelial cells via paracellular transmigration, particularly if TJ are compromised. The parasite may also directly infect retinal endothelial cells, replicating within them and eventually breaching into retinal tissues.

Pathophysiologically, *T. gondii* preferentially infects retinal Müller glial cells and retinal pigment epithelial cells, altering their function and inducing a robust immune response [106,107]. The immune response in ocular toxoplasmosis is complex, involving cytokines such as IFN-γ and IL-17, which play roles in controlling the parasite proliferation but can also contribute to tissue damage if dysregulated [105]. The immune-privileged status of the eye complicates the pathophysiology, as systemic immune responses do not directly translate to ocular immunity. While significant progress has been made in understanding the pathogenesis and immune responses in ocular toxoplasmosis, further research is needed to develop more targeted therapies and to fully elucidate the mechanisms underlying chronic infection and recurrent disease.

### Uncovering new models for studying transmigration of *T. gondii*: modeling biological barriers and organs

Organ-on-a-chip and microfluidic systems have emerged as valuable tools for studying the complex interactions between *T. gondii* and host tissues. These systems offer a more physiologically relevant environment compared to traditional *in vitro* models such as 2D monolayers, allowing for the investigation of parasite behavior and host responses in a controlled environment that mimics villous architecture and fluid shear [108].

A microfluidic model for *in vitro* acute *T. gondii* infection and transendothelial migration mimics human vasculature, enabling the study of *T. gondii* interactions with endothelial cells. This three-dimensional system supports the parasite’s lytic cycle *in situ* and allows real-time monitoring of growth, paracellular and transcellular migration, and transmission of the parasite across endothelial barriers [109]. It provides a controlled environment to investigate *T. gondii* adhesion and migration, shedding light on systemic dissemination mechanisms, including spread to the brain and eyes. Additionally, the model simulates physiological shear stress, which enhances tachyzoite motility and influences endothelial adhesion and migration, offering insights into *T. gondii* navigation and barrier breach during infection [110].

**Fig 4:**
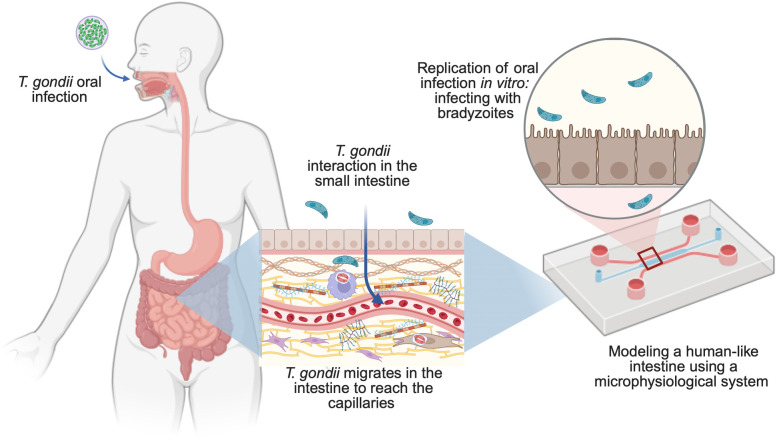
Studying *Toxoplasma gondii* infection by using microphysiological devices: modeling a human intestine. The microphysiological system simulates the intestinal environment, allowing the investigation of parasite early infection, stage conversion, migration through intestinal epithelial cells, its impact on the intestinal barrier, and subsequent spread to other tissues. This model enables the analysis of immune responses and therapeutic interventions. Figure created with BioRender.com.

Furthermore, using a microphysiological system that replicates the human intestinal environment (gut-on-a-chip) has allowed to observe the earliest interactions between *T. gondii* and intestinal tissue—events that are virtually impossible to capture in the murine model (Fig 4) [40]. In mice, sparse intestinal parasite loads prevent imaging during the first 24–48 h of infection; useful views emerge only after day 3. The gut-on-a-chip overcomes this by recreating a 3D epithelium that is optically accessible, letting to watch, parasite-enterocyte contact and migration into lamina propria–like stroma in real time—thus providing insights unattainable in the animal model while using human cells lines [40]. This model allowed a detailed analysis of *T. gondii* ingress, replication, and stage conversion, particularly the transition from bradyzoites within the cysts to tachyzoites essential for systemic spread. By incorporating collagen matrices to mimic the intestinal stroma, the system simulates *T. gondii* migration and replication dynamics. It also enables observation of the parasite’s migration through the intestinal barrier, providing insights into how *T. gondii* breaches the gut epithelium [40].

These advanced models, represent significant progress in the study of *T. gondii*, offering new opportunities to unravel the complexities of its biology and host interactions. They provide a platform for more detailed investigations into the parasite’s life cycle, host-pathogen interactions, and the development of novel therapeutic strategies.

## Methodology

The literature review was conducted to examine *Toxoplasma gondii* dissemination, migration, and interactions with biological barriers, focusing on the early gut/intestine interactions and the migration of immunoprivileged organs. Searches were performed in PubMed, OpenEvidence, and other scientific databases using keywords and small sentences such as *Toxoplasma gondii* and*: dissemination*, *migration*, *extracellular matrix*, *biological barriers*, *ocular toxoplasmosis*, *immunoprivileged organs*, *human toxoplasmosis, cancer, metabolic syndrome, encephalitis, drug delivery,* early *gut/intestine interaction, paracellular migration, paracellular/transcellular migration, organ-on-a-chip and microphysiological/microfluidic systems.*

Inclusion criteria encompassed original research articles, reviews, and peer-reviewed studies published in the last two decades, while studies not related to the topics or without full-text access were excluded. Data were extracted on experimental models, findings related to parasite migration and interactions with extracellular matrix components, and mechanisms involved in crossing biological barriers, particularly the gut and ocular tissues. This approach helped identify knowledge gaps and future research directions in *T. gondii* pathogenesis.

**Key learning points**

- *Toxoplasma gondii* is a widespread parasite causing both acute and chronic infections, particularly in the CNS and muscles.
- *T. gondii* infection in the CNS can lead to neuroinflammation and behavioral changes, including anxiety and depression.
- The parasite spreads across biological barriers, using mechanisms like the “Trojan horse” strategy, with paracellular direct migration pathways also proven.
- Advanced models like microfluidic and organ-on-a-chip systems help study *T. gondii*’s interactions with host tissues and systemic dissemination.
- *T. gondii*’s ability to cross biological barriers, including the blood–brain barrier, plays a significant role in its pathogenesis.

**Top five papers**

1. Dubey JP, Lindsay DS, Speer CA. Structures of *Toxoplasma gondii* tachyzoites, bradyzoites, and sporozoites and biology and development of tissue cysts. Clin Microbiol Rev. 1998;11: 267–99.
2. Barragan A, Sibley LD. Transepithelial migration of *Toxoplasma gondii* is linked to parasite motility and virulence. J Exp Med. 2002;195: 1625–33. https://doi.org/10.1084/jem.20020258
3. Gregg B, Taylor BC, John B, Tait-Wojno ED, Girgis NM, Miller N, et al. Replication and distribution of *Toxoplasma gondii* in the small intestine after oral infection with tissue cysts. Infect Immun. 2013;81: 1635–43. https://doi.org/10.1128/IAI.01126-12
4. Olivera GC, Ross EC, Peuckert C, Barragan A. Blood–brain barrier-restricted translocation of *Toxoplasma gondii* from cortical capillaries. Elife. 2021;10. https://doi.org/10.7554/eLife.69182
5. Ramírez-Flores CJ, Hryckowian ND, Gale AN, Babatunde KA, Lares M, Beebe DJ, et al. Modeling *Toxoplasma gondii*-gut early interactions using a human microphysiological system. Ewald S, editor. PLoS Negl Trop Dis. 2025;19: e0012855. https://doi.org/10.1371/journal.pntd.0012855

## Abbreviations

**:**
AJ: adherens junction
BBB: blood–brain barrier
BFD1: bradyzoite-formation deficient 1
BRB: blood–retinal barrier
CNC,: central nervous system;
FAK: focal adhesion kinase
PDAC: pancreatic ductal adenocarcinoma
RFLP: restriction fragment length polymorphism
TEER: transepithelial electrical resistance
TIMP-1: tissue inhibitor of metalloproteinases-1
TJ: tight junction
